# Identification of Mutations in Antimalarial Resistance Gene *Kelch13* from *Plasmodium falciparum* Isolates in Kano, Nigeria

**DOI:** 10.3390/tropicalmed5020085

**Published:** 2020-05-27

**Authors:** Umar F. Abubakar, Ruqayya Adam, Muhammad M. Mukhtar, Abdullahi Muhammad, Adamu A. Yahuza, Sulaiman S. Ibrahim

**Affiliations:** 1 Laboratory Department, Public Health and Diagnostic Institute, Yusuf Maitama Sule University, Kwanar Dawaki, PMB 3220 Kano, Nigeria; farouqabu@nwu.edu.ng; 2 Department of Biological Sciences, Faculty of Sciences, Federal University Dutsinma, PMB 5001 Katsina, Nigeria; radam@fudutsinma.edu.ng; 3 Department of Biochemistry, Bayero University, PMB 3011 Kano, Nigeria; muhammadmahemukhtar@gmail.com (M.M.M.); Abdullahi.Muhammad@lstmed.ac.uk (A.M.); 4 Vector Biology Department, Liverpool School of Tropical Medicine (LSTM), Liverpool L3 5QA, UK; 5 Department of Medical Microbiology and Parasitology, Faculty of Clinical Sciences, Bayero University, PMB 3011 Kano, Nigeria; ayadamu.mpc@buk.edu.ng

**Keywords:** artemisinin combination therapy, resistance, SNP, mutation, *kelch13*, *P. falciparum*, malaria

## Abstract

Malaria control relies on first-line treatments that use artemisinin-combination therapies (ACT). Unfortunately, mutations in the *plasmodium falciparum* *kelch13* gene result in delayed parasite clearance. Research on what is causing ACT failure is non-existent in northwestern Nigeria. Thus, the presence of mutations in *kelch13* in *P. falciparum* isolates from Kano, Nigeria was investigated in this study. Microscopic examination of 154 blood samples obtained from patients revealed a high prevalence of *P. falciparum* infection (114 positive individuals, slide positivity rate = 74.03%). The 114 patients were administered Cartef^®^ (ACT) and out of the 50 patients that returned for the 14-day follow up, 11 were positive for *P. falciparum* (slide positivity rate = 22%). On day 0, 80 samples out of 114 and 11 samples on day 14 (91 out of 125 microscopy-positive samples) were positive with *Plasmodium* according to the PCR of *cytochrome oxidase* I, which corresponds to 72.8%. A fragment of the *kelch13* gene encompassing the propeller domains was sequenced in 49 samples, alongside samples of the susceptible strain *pf_*3D7. Low polymorphism was observed, suggesting a lack of selection on this gene, and only six mutations (Glu^433^Gly, Phe^434^Ile, Phe^434^Ser, Ile^684^Asn, Ile^684^Thr and Glu^688^Lys) were found. The epidemiologic impact of these mutations and their potential role in ACT resistance needs to be investigated further.

## 1. Introduction

Malaria still takes the lives of approximately 405,000 people every year, 93% of these are in Africa, of which Nigeria alone accounts for 25% [1]. Malaria control relies heavily on vector control and administration of antimalarial medicines [2]. The use of antimalarials and vector control has contributed to a significant reduction in the malaria burden in Africa by about 40% from 2000 to 2015 [3]. Starting from 2005, the World Health Organization (WHO) recommended artemisinin combination therapy (ACT) as a first-line therapy for uncomplicated *P. falciparum* malaria, a policy that was adopted by many endemic countries [4]. Nigeria first adopted ACT drugs as a first-line treatment of uncomplicated *P. falciparum* in 2005 [5]. Currently, there are no alternative, fully effective first-line therapies available to replace ACT should artemisinin fail globally [6]. Unfortunately, ACT resistance, which was first discovered in the Greater Mekong Sub-Region [7] and is now widespread across the world [4], is threatening the progress made in malaria control. Several mutations, including N458Y, Y493H, R539T, I543T, and C580Y in the propeller domain of the *kelch13* gene of *Plasmodium falciparum* were found to be associated with delayed parasite clearance following artemisinin treatment, as observed in Southeast Asia [8,9]. The *kelch13* encodes 726 amino acids with a broad-complex, tram track, bric-a-brac/poxvirus and zinc finger (BTB/ POZ) domain and a C-terminal 6-blade propeller domain [8], where most mutations linked to delayed parasite clearance are found. However, most of the polymorphisms of the *kelch13* gene that have been described in a few studies in Africa vary from those observed in Southeast Asia [10], with radical mutations common in Southeast Asia and the African mutations not undergoing strong selection [11]. Therefore, it is still not understood whether the mutation of this gene in the African parasite population correlates with selection induced by antimalarial usage [12]. Several studies have been conducted in African countries to establish the presence of the *kelch13* mutations, and have led to the discovery of several mutations, for example, the non-synonymous mutations (M472I; Y558C; K563R; P570L; P615S) discovered in Niger [13], a novel mutation, R622I discovered in Ethiopia [14], 15 non-synonymous mutations in isolates from Senegal [15], and the P553L mutation reported in Mali, Kenya, and Malawi [16]. However, these mutations have not been linked to delayed parasite clearance. The main mutations linked to delayed parasite clearance and artemisinin resistance in the *kelch13* gene, e.g., Y493H, C580Y and Y493H occur at a very low frequency across Africa [11]. Indeed, a recent metadata analysis by the WorldWide Antimalaria Resistance Network (WWARN) reported that *kelch13* mutations in African sites remains at very low prevalence, generally below 3%, and that there is still no evidence of slow-clearing parasites or selection for mutant parasites [17]. However, the emergence of indigenous artemisinin-resistant *P. falciparum* has been documented in Africa [18] and a mutation, A578S (observed at low-frequency) has been associated with prolonged parasite clearance in a study conducted in Uganda [19].

Recently, a study in southeastern Nigeria reported four synonymous substitutions at the propeller domain of the *kelch13* gene that do not result in amino acid substitution and are not associated with delayed parasite clearance [20]. These types of studies on polymorphism in the *kelch13* gene are hard to come by in northwestern Nigeria, where the bulk of the country’s population lives. To contribute to malaria elimination efforts in northern Nigeria, single nucleotide polymorphisms (SNPs) and mutations in a *kelch13* propeller gene in *P. falciparum* isolates from Kano was investigated. This established the presence of four mutations that have not been described elsewhere, two of these were from individuals that returned on the 14-day follow-up with infection.

## 2. Materials and Methods

### 2.1. Sample Collection

Patients (18–56 years, n = 154) presenting with malaria at Murtala Muhammad Specialist Hospital and Nassarawa Hospital in the Kano metropolis were recruited for this research. Younger individuals and pregnant women in their first trimester were excluded. Ethical clearance (MOH/Off/797/T.I./402) for sample collection was provided by the Ethics Sub-Committee of Health Operational Research Unit, Ministry of Health, Kano State, Nigeria.

Blood samples were collected on 24–25 August 2018 by standard venipuncture without undue pressure, either on the arm or syringe. Thick and thin blood films were prepared on clean, grease-free glass slides, and 3 ml of the whole blood was dispensed into ethylenediaminetetraacetic acid (EDTA) anticoagulated containers. The blood samples were screened for the presence of malaria parasite using Geimsa staining microscopy [21] at both of the hospitals. Samples were then transferred on ice to the Department of Biochemistry, Bayero University, Kano (BUK) for molecular analysis.

### 2.2. Drug Treatment and Follow Up

On the day of the first sample collection (day 0), 114 patients were identified as infected with *P. falciparum* using thick and thin blood film microscopy. Microscopic examination of slides from the rest of the patients (forty individuals) revealed no infection of any of the four major human malaria parasites. The 114 patients infected with *Plasmodium* were provided with ACT, Cartef^®^ (GB PHARMA, United Kingdom), an Artemether/Lumefantrin (80 mg/480 mg taken twice daily). The dangers of non-compliance with the drug regimen was explained in detail to all patients. Parasite clearance was evaluated after 14 days of drug therapy in the 50 individuals that returned for follow-up, by blood collection and microscopy (conducted independently in the hospitals and BUK). The positive samples from the microscopy at day 0 (114 individuals) and those from the 14-days after treatment (50 individuals returned and 11 of them were still infected with *P. falciparum*) were used for downstream molecular analyses.

### 2.3. DNA Extraction and Confirmation of Plasmodium Falciparum Infection Using PCR

DNA was extracted from the whole blood of 125 patients who were positive for *P. falciparum*. These included all 114 samples collected at day 0, and the 11 positive samples from follow up. DNA isolation was carried out using the QIAamp^®^ DNA Mini Kit (QIAGEN, Hilden, Germany) according to the manufacturer’s instructions. DNA pellet from an antimalarial susceptible parasite strain, 3D7 was provided by Dr Janet Storm from the Parasitology Department, LSTM, UK and used alongside the field samples for DNA extraction. The DNA was eluted in 100 µL of nuclease-free water and its concentration measured using the Qubit 4.0 fluorometer (Invitrogen, Massachusetts, USA). Samples were stored at −20 °C.

Due to the drawbacks of *Plasmodium* microscopy (false positives associated with artifacts and contaminants) [21,22], the direct PCR method as described by Echeverry et al. [23] was used to confirm infection. Primers, CoxI-F 5’-agcggttaacctttctttttccttacg−3’ and CoxI-R (5′-agtgcatcatgtatgacagcatgtt−3′) targeting the *cytochrome oxidase* I (*COX* I) subunit of *Plasmodium* species was used for the PCR. Briefly, 1 μL each of genomic DNA, 1.5 μL of 10× TaqA Buffer, 0.4 μM (0.5 μL) each of forward and reverse primers, 0.63 mM (0.375 μL) of MgCl_2_, 0.4 mM (0.24 μL) of dNTP mixes, 0.2 μL of *Taq* DNA polymerase and 10.66 μL ddH_2_0 were reconstituted into a final volume of 15 μL. Amplification was carried out using the following cycling conditions: initial denaturation at 95 °C for 3 min, followed by 35 cycles each of 1 min at 94 °C (denaturation), 1 min at 62 °C (primer annealing), and 1 min at 72 °C (extension). This was followed with a 10 min final extension at 72 °C. PCR products were separated in a 1.5% agarose gel stained with pEqGREEN (GeneOn, Leicestershire, England) and visualized using Ingenius 3 Gel Doc (Syngene, Cambridge, UK).

### 2.4. Amplification of Propeller Domains of the Kelch13 Gene and Column Purification

The DNA samples were used to amplify a fragment of the *kelch13* gene encompassing the propeller domain, based on the nested PCR protocol of Ariey et al. [8] with modifications in the kelch-in primers and thermocycling conditions. In the first round PCR, primers kelch-out-F (5′-gggaatctggtggtaacagc−3′) and kelch-out-R (5′-cggagtgaccaaatctggga−3′) were used. PCR was carried out in a 20 μL final volume comprised of 2 μL of the genomic DNA, 10 μL of a GoTaq master mix (Promega, Wisconsin, USA) containing optimized buffer, MgCl_2_ and dNTP mixes; 1 μL each of forward and reverse primers and 6 μL ddH_2_0. Thermocycling conditions were initial denaturation at 95 °C for 1 min, followed by 35 cycles each of 20 sec at 95 °C (denaturation), 20 sec at 57 °C (primer annealing), 1.5 min at 60 °C (extension). This was followed with a 3 min final extension at 60 °C. PCR products were separated in 1.5% agarose gel stained with pEqGREEN and visualized for bands. For nested PCR, primers kelch-in-F2 (5′- cataccaaaagatttaagtgaaagtgaagc−3′) and the kelch-out-R (above) were used. PCR was carried out in a final volume of 20 μL comprised of 2 μL of the genomic DNA, 10 μL of master mix (Promega, Wisconsin, USA), 1 μL each of forward and reverse primers and 6 μL of ddH_2_0. Amplification was carried out using the following conditions: initial denaturation at 95 °C for 1 min, followed by 35 cycles each of 20 sec at 95 °C (denaturation), 20 sec at 57 °C (primer annealing), 1 min at 60 °C (extension). This was followed with a 3 min final extension at 60 °C. PCR products were separated in a 2% agarose gel stained with pEqGREEN and examined for bands. The nested PCR products were purified using the QIAquick^®^ PCR Purification Kit (QIAGEN, Hilden, Germany) and DNA eluted in 30 µL of nuclease-free water.

### 2.5. Cloning of the Kelch13 Fragment and Sequencing

The purified nested products were ligated into pJET1.2 (CloneJET PCR Kit, ThermoFisher Scientific, UK) vector according to the manufacturer’s instruction. Ligated products were transformed into DH5α *E. coli* cells (Promega, Wisconsin, USA) by mixing 4 µL of the purified nested products to 40 µL of the cells pre-chilled on ice. This was incubated on ice for 30 min followed by heat shocking for 45 sec at 42 °C. Transformants were returned to ice and chilled for 2 min before 950 µL of S.O.C medium was added. Transformants were incubated for 1 h at 37 °C and 200 rpm. Then, 100 µL of the transformants were streaked onto LB plates containing 100 mg/mL ampicillin and incubated at 37 °C overnight. Colonies that had grown in the plates were individually picked and diluted in 20 µL ddH_2_0 and used for colony PCR. Primers, pJET1.2-F (5’-cgactcactatagggagagcggc−3’) and pJET1.2-R (5-aagaacatcgattttccatggcag−3’) were used in PCR to identify positive colonies. 1.5 µL of 10× Taq A buffer, 2 µL of dNTP mixes, 0.75 µL of 2 mM MgCl_2_, 0.4 µL each of above primers, 0.1 µL of KAPA*Taq* polymerase and 13.9 µL of ddH_2_0 were constituted into a final volume of 19 µL. Then, 1 µL of colony suspended in ddH_2_0 was added to this and PCR was carried out with the following conditions: initial denaturation at 95° C for 3 min, followed by 35 cycles each of 30 sec at 94 °C (denaturation), 30 sec at 60 °C (primer annealing), 1.5 min at 72 °C (extension). This was followed with 5 min final extension at 72 °C. PCR products were separated in a 1.5 % agarose gel as described above. Positive colonies were mini-prepped overnight. Positive colony (4 µL) in ddH_2_O and 4 µL of 100 mg/mL ampicillin were added into a 15 mL tube containing 6 ml of LB medium. Tubes were incubated at 37 °C and 200 rpm for 14 h and 4.5 mL of overnight culture pelleted at 13000 rpm for 10 min. Plasmid preparation was carried out using the QIAprep^®^ Spin Miniprep Kit (QIAGEN, Hilden, Germany) and the plasmid concentration was measured using a Nanodrop spectrophotometer (Thermo Fisher Scientific, Massachusetts, USA). Plasmids were sequenced using the pJET1.2-F and pJET1.2-R primers mentioned above.

### 2.6. Analysis of Genetic Variability of Kelch13

Polymorphism analysis of sequences was carried out through manual examination of the sequence traces using Bioedit version 7.2.3.0 [24] and/or nucleotides/amino acid differences from multiple sequence alignments with the CLC sequence viewer v7.6 (http://www.clcbio.com/). Genetic parameters such as the number of haplotypes (h) and its diversity (Hd), the number of polymorphic sites (S) and nucleotide diversity (π) were computed using DnaSP 5.10 [25]. Different haplotypes were compared by constructing a maximum likelihood phylogenetic tree, using MEGA 6.06 [26].

## 3. Results

### 3.1. Malaria Slide Positivity Rate

From 154 suspected malaria patients recruited on day 0, 114 were positive for *P. falciparum* (74.03%) (Table 1). Analysis of samples from 50 patients who returned for the 14-day follow up (out of the 114 patients that were given Cartef^®^) found that 11 of these patients were still infected with *P. falciparum* (day−14 slide positivity = 22.0%). Infection in these samples was confirmed with PCR and they were then used along with the initial 114 patients from day 0, making a total of 125 samples for downstream molecular analyses.

### 3.2. Cytochrome Oxidase III PCR Confirmation of Plasmodium Infection

**Figure 1:**
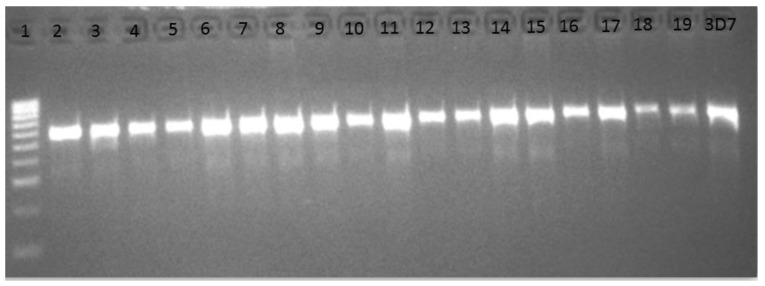
*Cytochrome oxidase* I (*COX* I) PCR confirmation of Plasmodium infection. Lane 1 = Molecular ladder (Hyper ladder IV: 50–1013 bp, Bioline), lanes 2–19 = 540 bp representing *COX* I gene of malaria parasite. Lane 20 is the band from the 3D7 reference susceptible strain.

Out of the 125 samples used for PCR, only 91 samples (72.8%) were confirmed as positive for *P. falciparum*/*P. vivax* (Figure 1).

### 3.3. Amplification and Cloning of the Kelch13 Fragment

**Figure 2:**
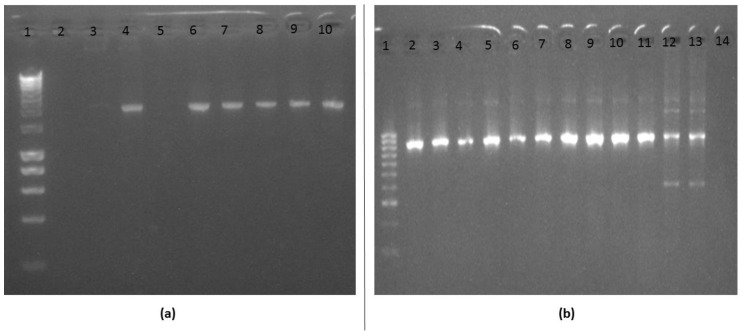
Agarose gel of PCR for amplification of kelch13 fragments. (**a**) first round PCR, lane 1 = molecular ladder (Hyper ladder 1200 bp–10 kb, Bioline); lanes 3, 4, 6, 7, 8, and 9 represent samples from malaria-infected individuals with 2097 bp fragment. Lane 10 = 3D7 reference susceptible Plasmodium strain; lane 2 is negative control with no DNA and lane 5 is example of sample that failed; (**b**) nested PCR, lane 1 = molecular ladder (Hyper ladder IV, 50–1013 bp, Bioline), lanes 2–12 = band size of 849 bp, corresponding to kelch13 propeller domain of Plasmodium isolates; lane 13 = 3D7 and lane 14 is negative control with no DNA. Non-specific bands were seen in the samples in lanes 12 and 13. Note: the samples in panel 2a and 2b are not correlated.

In first round PCR, DNA from 91 samples was used to successfully amplify fragment of *kelch13* gene, with PCR products of 2097 bp (Figure 2a). These include 80 of the 82 day 0 samples that were positive for Plasmodium from the PCR, plus the DNA from 11 follow-up samples.

From nested PCR, 2849 bp fragments corresponding to nucleotides 1281–2129 (which covers almost the entire sequence of the Broad complex tram track bric a brac (BTB) and six blades of propeller domains, codons 427–709) were amplified successfully in 77 samples (Figure 2b). Because of the non-specific bands that were observed in some samples, e.g., in lanes 12 and 13 in Figure 2b, the 77 nested PCR products were purified and successfully cloned into *E. coli* DH5α to confirm sizes using PCR. Positive colonies were mini-prepped and 50 samples were sequenced.

### 3.4. Pattern of Genetic Variability of the Kelch13 Fragment

Out of the 77 purified nested PCR products, 50 were successfully sequenced. These comprised 49 field samples and a fragment amplified from the pf_3D7. The 49 field samples comprised seven samples from the follow-up individuals and 42 samples from day 0. Analyses of all sequences revealed eight mutations compared to the pf_3D7 sequences (Table 2). These sequences have been deposited into the Genbank with accession numbers: MT263314-MT263363. Two isolates; MM_12B and MM_83B from the 14-day follow up harbor Glu**^433^**Gly and Glu**^688^**Lys, mutations, respectively. These mutations are not seen in the other day 0 isolates or the other five samples from follow up. In addition, the other four non-synonymous mutations include Phe**^434^**Ile obtained from day 0 isolates MM_31 and MM_21, as well as Phe**^434^**Ser, Ile**^684^**Asn, Ile**^684^**Thr, which were present only in the day 0 isolates MM_114, MM_10 and NSR_59, respectively.

**Figure 3:**
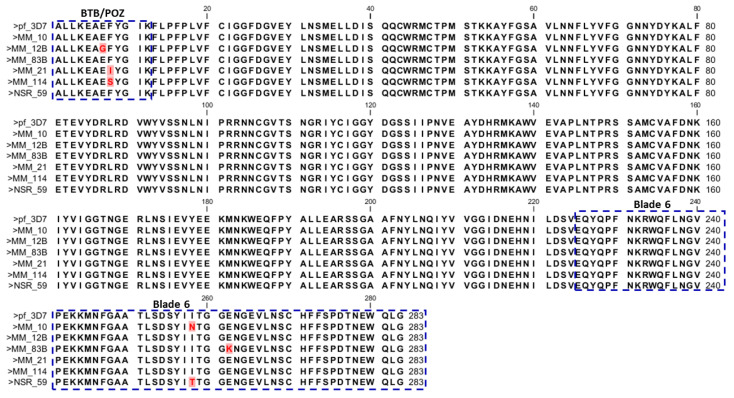
Comparison of amino acid sequences from field isolates with the amino acids of Pf_3D7 sequence. Residues with mutations are in red and highlighted. MM_83B and MM_12B are sequences from isolate of patients that responded to the 14-day follow up and were still infected with *Plasmodium*. Nucleotide positions were compared to the description in the literature: 350–442, BTB/POZ, 442–475; blade 1475–527; blade 2527–574; blade 3574–614; blade 4614–666; blade 5666–727, blade 6. Different amino acids are in red and highlighted in pink.

Six of these mutations are non-synonymous (NS): Glu**^433^**Gly, Phe**^434^**Ile, Phe**^434^**Ser, Ile**^684^**Asn, Ile**^684^**Thr and Glu**^688^**Lys. The first three mutations are in the BTB/POZ domain and the last three are in the 6th blade of the propeller domain (Figure 3).

From the 49 samples sequenced, the kelch13 was discovered not to be highly polymorphic. It has nine haplotypes (Table 3) with haplotype diversity, Hd of only 0.336. The sequences possess eight polymorphic sites (S); two of which were synonymous and six led to amino acids substitution. A neutrality test of all the sequences revealed Li and Fu’s D* as negative and statistically significant, which indicates an excess of singleton mutations. The Tajima D statistic, on the other hand shows a low frequency of polymorphism. Overall, the presence of a dominant haplotype and very low diversity, with most mutations being non-synonymous suggests that this gene is either probably undergoing selection, or these mutations are just rare and endogenous.

**Figure 4:**
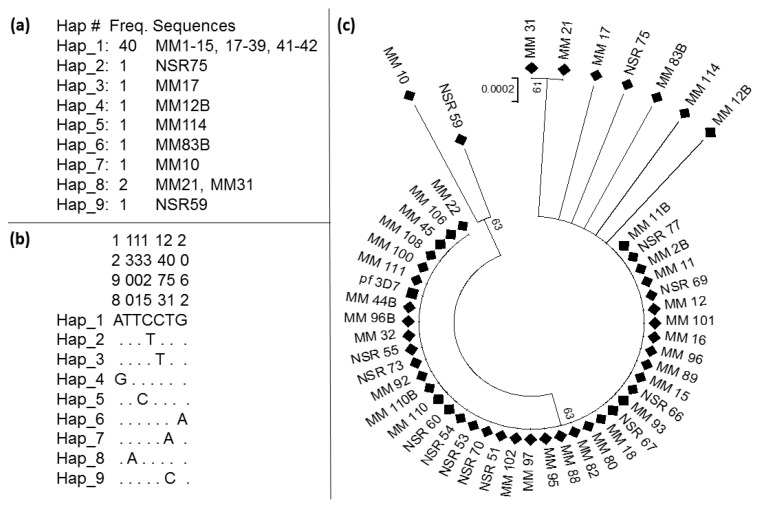
Genetic variability of fragments of kelch13 gene. (**a**) Summary of haplotype frequencies; (**b**) nucleotide substitution positions; and (**c**) a maximum likelihood phylogenetic tree of sequences with all field sequences presented in a diamond shape and the sequence from pf_3D7 in a block square.

A predominant haplotype, which had the largest frequency was observed (Figure 4a). This haplotype 1 is comprised of 40 sequences out of 49. It was followed by haplotype 8 with two sequences, while the rest of the haplotypes have only one sequence each. Figure 4b presents the position of the nucleotide substitution, respectively, with respect to the predominant haplotype 1.

To establish genetic distances, a phylogenetic tree was constructed with the 49 sequences successfully sequenced and a sequence of pf_3D7 (Figure 4c). Sequences cluster according to the presence of mutation, with the sequences harboring mutations clustering away from sequences of the Hap_1 and that of the pf_3D7.

## 4. Discussion

This study investigated the presence of polymorphism in the *kelch13* gene using *P. falciparum* isolates from northern Nigeria. Eight polymorphic sites were discovered in the propeller region of this gene, with six of them leading to amino acids substitution. However, none of the four most implicated mutations associated with ACT resistance (Y493H, R539T, I543T, or C580Y) [8] were seen in the field isolates from Kano. Indeed, the absence of these mutations has been reported previously in southeastern Nigeria and other African countries, including Niger, Cameroon and Benin, that share borders with Nigeria [20,27]. However, several other mutations exist across the propeller domain of the *kelch13* gene in sub-Saharan African *P. falciparum* isolates, with 22 major non-synonymous mutations already described. These include A557S, V566I, A569T, S576L, A578S, L589I, with the A578S being the most common [16,28,29]. None of these mutations were found in the field isolates investigated in this study. In Senegal, N554H, Q613H and V637I were reported [30] and recently in the Niger Republic, five mutations (M472I, Y558C, K563R, P570L and P615S) were also described in the propeller domain of *kelch13* gene [27]. However, none of these mutations was detected in any of our isolates, despite the geographic closeness between the Niger Republic and Kano, in northern Nigeria. Several other mutations observed in other African countries include the most frequent mutation in Mali, F446I [31] and a few synonymous mutations reported in Burkina Faso [32]. None of these mutations were found in samples from Kano. The mutations observed in the Kano isolates may be unique to the Sudan/Sahel of northwest Nigeria. The high heterogeneity in the *kelch13* gene mutations across sub-Saharan Africa suggests lack of selection in this gene. The results of microscopy strengthen previous observations of *P. falciparum* being the primary parasite responsible for malaria in northern Nigeria. Previous studies in Nigeria show similar results, for example, in southwestern Nigeria, where malaria parasite positivity was found among 61.1% of the study population [33]. Similarly, a 73.39% prevalence was also documented in another study in southeast of Nigeria [34].

## 5. Conclusions

This study confirmed a high prevalence of falciparum malaria in Kano, northwestern Nigeria. As established in various previous studies, it was found that microscopy could lead to false positives and its result should be treated with caution. The *kelch13* gene in *P. falciparum* isolate from Kano carries some novel mutations that should be further studied to establish their epidemiologic impact on antimalarial resistance.

## Figures and Tables

**Table 1:** Summary of malaria parasite microscopy.

Day of Treatment	No. of Positive	No. of Negative	Total No. of Samples
Day 0	114 (74.0%)	40 (26.0%)	154 (100%)
Day 14 (Follow Up)	11 (22.0%)	39 (78.0%)	50 (100%)
Total	125	79	204

No. = Number. Day 0 represents the day of malaria diagnosis prior to drug treatment, while day 14 is the 2-week follow up day after initiating drug treatment, to evaluate parasitemia after drug administration.

**Table 2:** Polymorphisms observed in the kelch13 fragments and amino acid substitutions.

Domain/ Propeller	Wild Type Codon	Polymorphic Site	Position (nt)	Observed Mutation	Sequence (s) with Mutation	Substitution Type	Day of Collection
BTB/POZ BTB/POZ	GAA TTT	GGA ATT	1298 1300	Glu^433^Gly Phe^434^Ile	1 (MM_12B) 2 (MM_31, MM_21)	NS NS	14th 0
BTB/POZ	TTT	TCT	1301	Phe^434^Ser	1 (MM_114)	NS	0
Blade 1	TTT	TTC	1325	Phe^442^Phe	1	S	0
Blade 2	TTT	TTC	1473	Phe^492^Phe	1	S	0
Blade 6	ATT	AAT	2051	Ile^684^Asn	1 (MM_10)	NS	0
Blade 6	ATT	ACT	2051	Ile^684^Thr	1 (NSR_59)	NS	0
Blade 6	GAA	AAA	2062	Glu^688^Lys	1 (MM_83B)	NS	14th

BTB/POZ; Broad complex tram track bric a brac/poxvirus zinc finger, nt; nucleotide; No.: Number; Glu: Glutamic acid; Gly: Glycine; Phe: Phenylalanine; Ile: Isoleucine; Ser: Serine; Asn: Asparagine; Thr: Threonine; Lys: Lysine.

**Table 3:** Summary statistics for polymorphism of fragment of kelch13 gene in P. falciparum isolates from Kano.

N	S	H	H_d_	Syn	Nonsyn	π (k)	D (Tajima)	D* (Fu and Li)
49	8	9	0.336	2	6	0.00043(0.364)	−2.18234^sig^	−3.67210 ^sig^

N; number of sequences; S, number of polymorphic sites; h, haplotype; H_d_, haplotype diversity; Syn, Synonymous mutations; Nonsyn, Non-synonymous mutations; π, nucleotide diversity (k = mean number of nucleotide differences); Tajima’s D and Fu and Li’s D statistics; ns, not significant; sig, significant.
